# Dietary Exposure to Food Contaminants of Pregnant Women in Northern Spain and Possible Effects on Fetal Anthropometric Parameters

**DOI:** 10.3390/toxics13050399

**Published:** 2025-05-16

**Authors:** Daniel Hinojosa-Nogueira, José Ramón Bahamonde, Marta Aguilera-Nieto, Beatriz Navajas-Porras, Verónica González-Vigil, José Ángel Rufián-Henares, Silvia Pastoriza de la Cueva

**Affiliations:** 1 Unidad de Gestión Clínica de Endocrinología y Nutrición, Laboratorio del Instituto de Investigación Biomédica de Málaga (IBIMA), Hospital Universitario de Málaga (Virgen de la Victoria), 29590 Málaga, Spain; 2 Facultad Padre Ossó, Clínica Universitaria INYPEMA, Universidad de Oviedo, 33008 Oviedo, Spain; jramon@facultapadreosso.es; 3 Hospital Reina Sofía, 31500 Tudela, Spain; marta1998_8@hotmail.com; 4 Department of Endocrinology and Nutrition, University Hospital Doctor Peset, Foundation for the Promotion of Health and Biomedical Research in the Valencian Region (FISABIO), 46017 Valencia, Spain; beatriz.navajas@fisabio.es; 5 Fundación Alimenta Tu Salud, 33208 Gijón, Spain; info@fundacionalimentatusalud.com; 6 Departamento de Nutrición y Bromatlogía, Instituto de Nutrición y Tecnología de los Alimentos, Centro de Investigación Biomédica, Universidad de Granada, 18071 Granada, Spain; spdelacueva@ugr.es

**Keywords:** food contaminants, pregnant women, dietary exposure, fetal anthropometry

## Abstract

A considerable number of organizations are working to improve food safety, with particular attention to vulnerable groups such as pregnant women due to the important influence of diet on fetal development. The aim of this study was to evaluate exposure to 11 food processing contaminants and their effects on maternal and fetal health. Online questionnaires during the first and third trimesters were used to analyze the intake and exposure to different food contaminants, estimated from the contaminants food database “CONT11”, in 84 pregnant women in Oviedo (Spain) and their influence on newborn anthropometric data. Exposure to certain contaminants, such as acrylamide or total polycyclic aromatic hydrocarbons, was found to have a significant impact on maternal and fetal health, particularly in relation to birth weight or head circumference. During the third trimester, pregnant women reported dietary improvement and reduced exposure to dietary contaminants. Identifying the foods and food groups that contribute most to exposure and the potential for health professionals will facilitate the development of basic exposure reduction strategies. This study is one of the few to assess the exposure of pregnant women to a wide range of contaminants and their impact on fetal health, providing a baseline for future research.

## 1. Introduction

A number of organizations, such as the World Health Organization (WHO), have a strong focus on the protection of the most vulnerable populations, such as pregnant women [1,2,3,4]. Numerous studies have investigated the role of nutrition during pregnancy, which has been identified as one of the most critical periods for preventing risks in childhood and adulthood, as it can influence embryonic development and be a link to later disease [1,3]. Therefore, ensuring food safety at this stage is of utmost importance.

Food can be a vehicle for pathogens, such as bacteria, viruses, or parasites, and many contaminants, such as pesticides, microplastics, heavy metals, or contaminants from food processing [5,6]. The United Nations Food and Agriculture Organization (FAO) and the European Food Safety Authority (EFSA) are developing databases and tools to assess dietary exposure to different food contaminants worldwide [7,8]. In this context, a database of contaminants from food processing, CONT11, has been developed in a previous study [9] providing information to assess the intake of up to 11 food contaminants: 5-Hydroxymethyl-2-Furfural (HMF5), pyrraline (PYRR), Amadori compounds (AMCOMP), furosine (FUROS), Acrylamide (ACRYL), Furan (FURAN), nitrates (NITRA), nitrites (NITRI), nitrosamines (NITRN), Benzopyrene (B(A)P), and total polycyclic aromatic hydrocarbon (TPAHC). Among the estimated food contaminants, some are produced by non-enzymatic chemical reactions during the heating and cooking of food, others are added during processing, and some may be present in food as a result of environmental contamination [10,11,12,13,14,15]. Some of these food contaminants have been described to be of concern for maternal and fetal health and development, giving rise to low birth weight, among other effects [11,16,17,18,19,20,21]. Therefore, understanding women’s exposure to food contaminants during pregnancy is important to predict how it might affect the health of their unborn children and to consider strategies to reduce the intake of these contaminants [20,22,23,24].

In light of these risks, health professionals, most notably midwives [25], are well-positioned to assume a pivotal role in the education of expectant mothers regarding the subject of dietary exposure to food contaminants [26]. Therefore, with all the above-stated information, the aim of this study was to assess the exposure of pregnant women in the north of Spain to different food contaminants and their possible effects on fetal anthropometric parameters, which will allow for more effective mitigation strategies to be designed.

## 2. Materials and Methods

### 2.1. Study Population

The study analyzed data from 84 pregnant women who completed food records and provided relevant anthropometric data during the first and third trimesters of pregnancy. Participants were enrolled online, were over 18 years of age, and were free of chronic diseases. All women gave birth in Oviedo, Asturias, Spain, and fetal anthropometric data were provided by the pregnant women after the initial postpartum consultation.

The study was a supplementary study derived from the European Stance4Health project [27], and informed consent was obtained from all participants in accordance with Spanish legislation and the ethical guidelines of the Declaration of Helsinki of the World Medical Association. Data on pregnant women were obtained and collected through an online questionnaire, including their age, height, weight in the first and third trimesters, tobacco consumption, and physical activity. Body Mass Index (BMI) was calculated using the formula (kg/m^2^). Gestational age and measurements of physical size, such as birth weight, height, and head circumference, were reported by the mothers and recorded according to standardized protocols and always under midwifery supervision [28,29,30,31].

### 2.2. Nutritional Information and Estimation of Contaminants

Dietary intake during pregnancy was assessed using a food frequency questionnaire (FFQ) developed and validated within the Stance4Health project [32]. The tool includes a total of 200 commonly consumed foods, which are categorized into distinct food groups such as “vegetables”, “fruits”, “fish and fish products”, and others [32]. In order to ascertain the most appropriate scale for the measurement of frequency of consumption, the data were reported on an incremental scale with nine levels (never or hardly ever, 1–3 times per month, 1 time per week, 2–4 times per week, 5–6 times per week, 1 time per day, 2–3 times per day, 4–6 times per day, 6 or more times per day). Each item was reported in terms of standard portion sizes, measured in grams or milliliters, and delineated according to common household portion sizes (plate, cup, tablespoon, teaspoon, glass, piece, slice). The FFQ was administered twice during pregnancy: once in the first trimester and again in the third trimester. Both procedures were conducted online.

Several contaminants can potentially be found in food. This study used the CONT11 database, which contains data on 11 contaminants. These contaminants may appear in food due to environmental contamination or intentional addition of additives. Further, the database accounts for contaminants that appear during the thermal processing of foods or during storage. CONT11 was developed following the standardized methodology for food composition databases (FCDB), which has been adapted to include chemical contaminants in food, treating them in a similar way to nutrients [9,33]. This approach allows contaminant concentrations to be expressed in comparable terms (milligrams or micrograms per 100 g of food), which facilitates their integration and analysis in dietary and exposure assessment contexts [9,33]. The CONT11 database was constructed from 34 sources, including scientific studies and official repositories, thus consolidating a robust resource for the analysis of the presence of contaminants in commonly consumed foods [9].

To determine the intake and exposure to food contaminants, data from the FFQ and the FCDB CONT11 were used. The methodology used in this study was based on other studies [9,34] where weighted averages of contaminant levels were used for those foods in the FFQ that had different concentrations depending on the cooking method. The intake of food contaminants was calculated taking into account the intake values of each food item and the concentration of each contaminant described in CONT11. Individual dietary exposure was determined by dividing the daily intake of the food contaminant by the body weight of pregnant women. Data were expressed as mg or µg contaminant/kg body weight/day. Finally, foods were grouped into 13 categories based on a standardized classification system, and the mean dietary exposure for each group was estimated. This allowed for the quantification of each food group’s relative contribution to the total dietary exposure to contaminants.

### 2.3. Data Statistical Analysis

The SPSS 26.0 statistical software was used to analyze data. The normality test Kolmogorov–Smirnov was performed on the data, followed by Spearman’s coefficient to evaluate their correlations between the different variables. Furthermore, linear regressions were conducted, adjusting for various covariates (maternal weight, age, energy intake, child sex, tobacco, and physical activity).

Variables related to intake of food contaminants were logarithmically normalized to visually demonstrate the difference in intake between the first and third trimesters of pregnancy, and these differences were formally tested for significance using the Mann–Whitney U test. The percentage difference in the intake of food contaminants between the first and third trimesters of pregnancy was also calculated. The level of significance was set at *p* < 0.05. Mean and standard deviation (SD) values were calculated for all variables. The Python 3.7 module was used to evaluate the association between the anthropometric parameters of newborns and the intake of food contaminants and to develop a heat map.

## 3. Results

### 3.1. Characteristics of the Study Population

The study analyzed the characteristics of 84 pregnant women and their newborns. An overview of the main features is provided in Table 1. The average age of participants was approximately 35 years, with an average weight of 68.2 kg and BMI of 23.7 kg/m^2^. The gestational age was 39.2 weeks, and 46.4% of the newborns were boys. With respect to the anthropometric parameters of the newborns, the mean birth weight was 3277 g ± 508 g. The height of the newborns was 50.3 cm, and the head circumference was 34.1 cm.

### 3.2. Distribution of Contaminant Intake by Food Group and Trimester of Pregnancy

**Figure 1:**
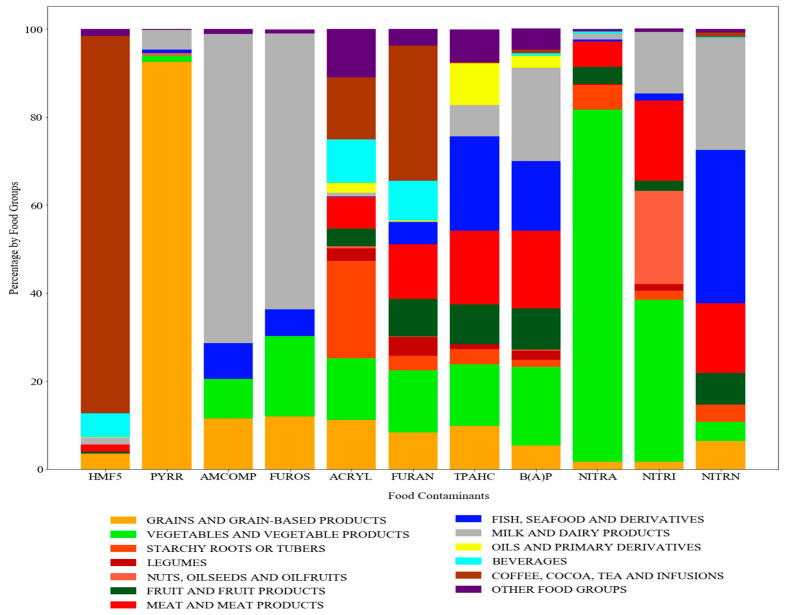
Percentage of food contaminants distributed by food group in relation to the food intake of pregnant women during both trimesters.

The intake of food contaminants was estimated using the FFQ adapted to CONT11. Figure 1 shows the percentages of different food groups consuming each contaminant.

The analysis showed that for the dietary intake of HMF5, the group of coffee, cocoa, tea, and infusions was responsible for 86% of the total exposure to this contaminant. The grains and grain-based products group was the major contributor (92%) to PYRR exposure. Milk and dairy products were the main sources of exposure to AMCOMP and FUROS, accounting for 70% and 63% of the respondents, respectively. The intake of ACRYL came from various products, but the most important were starchy roots or tubers (22%), with particular emphasis on fried products, other foods in which ultra-processed foods stand out (11%), and finally the group of coffee, cocoa, tea and infusions (14%). In the case of furan, its exposure came predominantly from the coffee, cocoa, tea, and infusions group, with a contribution of 30%.

The primary source of TPAHC and B(A)P exposure was the consumption of animal-based foods like meat, fish, and dairy products, which account for 45% and 54% of the intake, respectively. Additionally, TPAHC was also included in other food groups, such as oils, primary derivatives, and processed foods. B(A)P was also present in a significant proportion in vegetables and derivatives.

Regarding nitrate consumption, vegetables and plant products were the main culprits (80%). Nitrite intake was not only related to the consumption of vegetables and plant products (36%), but meat and meat products as well (18%); nuts, seeds, and oilseed fruits also played an important role (20%). Nitrosamines were predominantly found in fish, shellfish, and their derivatives (34.8%), and in other animal products (41%).

**Figure 2:**
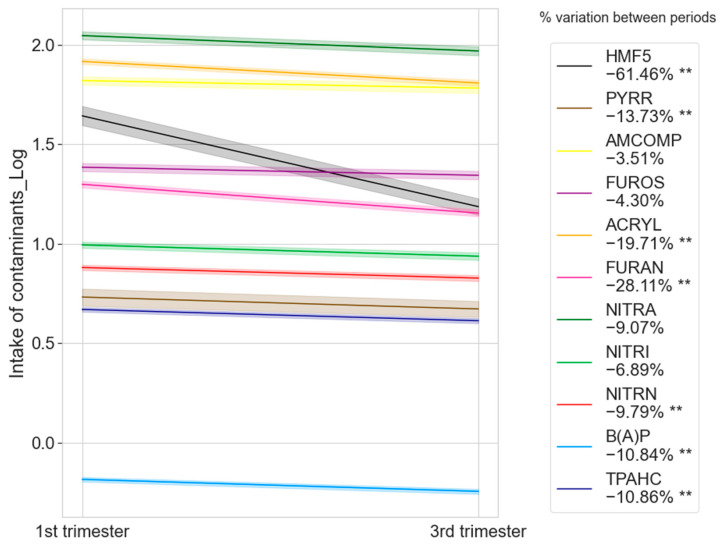
Percentage of the difference in the intake of food contaminants between the first and third trimesters of pregnancy. Differences between paired samples. ** Significant at the *p* < 0.05 level. Shaded bands represent the 95% confidence interval around that mean.

When comparing the intake of contaminants determined during the first and third trimesters of pregnancy, a reduction was observed for all contaminants during the third trimester. The reduction varied depending on the contaminant, ranging from 61% for HMF5 to 3.5% for AMCOMP. Figure 2 shows the contaminants that experienced statistically significant reductions.

### 3.3. Contaminant Intake and Exposure: Association with Fetal Anthropometric Parameters

The intake and exposure of 11 contaminants were estimated in 84 pregnant women using the Stance4Health FFQ. Table 2 presents the total intake of contaminants and exposure. Some contaminants, such as HMF, PYRR, and NITRA, showed high variability, whereas others were more stable.

**Figure 3:**
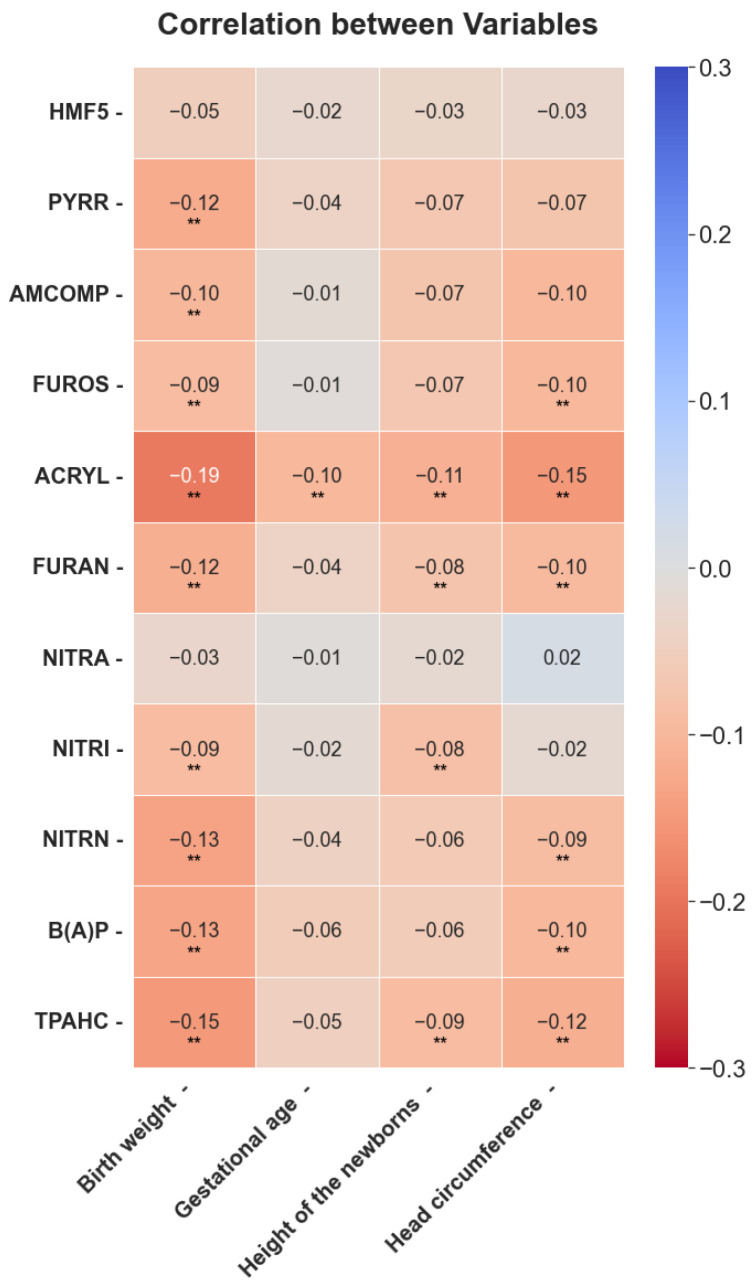
Heatmap with correlations between exposure to food contaminants and fetal anthropometry. Correlation between variables. ** Significant at the *p* < 0.05 level.

Based on evidence of exposure to food contaminants and their impact on maternal and fetal health, correlations between contaminant exposure and fetal anthropometric parameters were examined using Spearman analyses. Most contaminants had low negative correlations with specific anthropometric parameters (Figure 3).

For instance, birth weight was the most important parameter, and it decreased with higher contaminant exposure. PYRR, ACRYL, and TPAHC were identified as the contaminants with the most significant effects.

Based on these results, statistical models were developed to analyze the impact of contaminants on maternal and fetal health. However, the regression models demonstrated limited efficacy in predicting fetal anthropometry in relation to exposure to contaminants. For example, a decrease in birth weight was observed with an increase in ACRYL exposure; however, it was not significant (*p* = 0.07). Nevertheless, these models were found to be unable to explain a significant proportion of the observed results.

## 4. Discussion

### 4.1. Intake and Exposure to Food Contaminants and Their Relationship with Maternal and Fetal Health

Pregnant women constitute a vulnerable population, so it is essential to monitor their exposure to food contaminants, which can be potentially dangerous for fetal development and newborn health [17,18,35,36,37,38]. This study examined the dietary habits and levels of contaminants in the foods consumed by pregnant women.

The results of the evaluation of the intake of contaminants during the first and third trimesters of pregnancy (Figure 2) indicated that pregnant women significantly reduced the intake of all food contaminants during the third trimester. This reduction could be attributable to increased concerns about nutrition during pregnancy, especially during the third trimester [1,3]. In particular, the significant decrease in HMF5 exposure during the third trimester was observed as a result of a significant reduction in coffee consumption. This also explains the variability in exposure to some of the food contaminants studied, such as FURAN and ACRYL. Furthermore, the study indicates how certain eating habits increase or decrease exposure to specific contaminants, such as the consumption of vegetables, fried products, processed foods, or coffee [16,17]. One example was the variation in acrylamide intake among pregnant women in the Norwegian MoBa study due to products such as fried chips or nuts [17]. In contrast, foods of animal origin, including milk or meat, exhibited a higher regularity of consumption among pregnant women, which makes the exposure to some contaminants less susceptible to variability, such as FUROS or NITRI.

In this context, it should be noted that some of the food contaminants described in this study have not been well studied in pregnant populations, making comparisons difficult. For this reason, the intake of food contaminants studied in pregnant women (Table 2) was compared with the results of studies on other populations. The findings were consistent with those of other studies on the intake and exposure. For example, HMF5 intake was consistent with that reported in other studies on the Spanish population [39]. Concerning the levels of PYRR, AMCOMP, FUROS, or FURAN, the results of our study were lower than those described by other researchers [40,41,42]. In relation to ACRYL, which is one of the more studied food contaminants, the levels were in line with those found in another study of pregnant women, and do not exceed the exposure limits established by EFSA [18,22]. The results for TPAHC, particularly B(A)P, were consistent with the findings from previous studies [43,44]. However, it is imperative to consider the additional environmental exposure that would be added to the exposure reflected in our study [43,44]. Finally, the levels of nitrates, nitrites, and nitrosamines were comparable to, or lower than, those reported by other authors [45,46,47,48].

There are studies warning of the effect of some food contaminants (such as acrylamide or total polycyclic aromatic hydrocarbons) on birth weight, even promoting maternal obesity and the possibility of this being passed on to the offspring [11,16,17,18,20,21,36,37]. Moreover, their consumption can not only affect weight, but also can result in birth defects or even miscarriages [11,16,17,18,20,21,36,37]. Nitrates, nitrites, and nitrosamines are well known for their effects on oxygen deprivation in the newborn, and even fetal growth deficit has been observed after high intakes of nitrites from vegetable juices [35,38]. The remaining contaminants have not been studied extensively in pregnant women, and thus, there is not enough information about their potential effects on maternal and fetal health.

After considering the evidence on the effects of exposure on maternal-fetal health, it was important to explore the correlation between exposure to contaminants and fetal anthropometric parameters. Low inverse correlations were obtained (Figure 3) with respect to contaminant exposure and certain anthropometric parameters. In particular, birth weight was most affected by contaminant exposure. ACRYL was found as the contaminant more strongly associated with fetal anthropometry, confirming what has been described in other studies [11,18,36]; therefore, acrylamide should be one of the most significant contaminants to reduce. TPAHC, B(A)P, and FURAN were the next most relevant food contaminants after ACRYL, which supports what has been described in other studies, where high levels of these contaminants have been linked to low birth weight and prematurity [20,21,49,50]. In addition, nitrates were found to have the least effect on the weight of the fetus, confirming other studies describing certain benefits for maternal-fetal health due to their effects on blood pressure [19]. Additionally, ACRYL and FURAN had the greatest influence on gestational age.

### 4.2. Strategies to Mitigate Exposure to Food Contaminants in Pregnant Women

The assessment of the intake of food contaminants gives the opportunity to unravel what possible actions can be undertaken to mitigate and reduce their production, intake, and exposure. Organizations like the WHO or EFSA are working to reduce exposure to contaminants such as acrylamide [22,51,52,53]. Among the most important strategies to mitigate the intake and exposure of the food contaminants evaluated in this study are the following: i. reduce the consumption of food or food groups with the highest concentrations; ii. frequent replacement of the oil used for cooking; iii. use less heat-aggressive cooking methods; iv. reduce cooking temperatures; v use spices for cooking; vi. adjust parameters such as water activity or pH; vii. consider new methods of sterilization; and viii. increase pressure on the food industry to adopt measures to reduce these contaminants because of their health risks [24,51,54,55,56,57,58].

If careful attention on which food groups had a higher contribution to the exposure of these food contaminants throughout pregnancy was paid (Figure 1), a more reliable mitigation strategy could be found to reduce the exposure to different food contaminants for this specific population. In pregnant women, coffee consumption was the major contributor to HMF5 and FURAN exposure. Therefore, reducing coffee consumption could significantly reduce exposure to these contaminants. This is in line with what has been described in other studies, which suggest that coffee is one of the main sources of HMF5 [59,60,61]. Although grains and grain-based products are linked to exposure to almost all of the studied food contaminants, exposure to PYRR is the main concern [62]. It is necessary to identify the main contributors to this group and to explore possible food substitutes. Dairy products are a primary source of exposure to FUROS and AMCOMP. Therefore, an option could be to reduce the consumption of dairy products with aggressive sterilization methods [40,56]. Controlling NITRA and NITRI levels in drinking water and washing of vegetables would help to reduce the exposure to these contaminants [19,63]. The intake of meat and fish has a significant impact on the exposure to NITRI, NITRN, TPAHC, and B(A)P [15,48,64,65,66,67]. By reducing the consumption of smoked foods, processed meats, and foods with additives derived from these food contaminants, their exposure could be reduced. In addition, avoiding direct contact with flames during cooking or not reusing oils for frying at high temperatures would also mitigate the appearance of TPAHC and B(A)P [20,68]. Finally, ACRYL is associated with the consumption of processed and fried products, in particular fried potatoes. Reducing the consumption of these foods or modifying their cooking methods would result in a decrease in its exposure [13,22,23,24,51]. Another mitigation strategy, such as increasing the intake of vitamins due to their protective effects [50], or using microwave irradiation methods, could reduce exposure to these food contaminants [41].

However, the implementation of these strategies requires commitment from all professionals involved in the care of pregnant women, including doctors and nurses [69]. In this context, midwives, as a primary source of guidance during pregnancy, assume a pivotal role [70]. Enhancing their training in these strategies could effectively reduce their exposure to food contaminants [71]. A notable example is the French National College of Midwives, which has developed guidelines for this purpose [72].

### 4.3. Limitations and Strengths of the Study

Food composition databases generally do not estimate the levels of food contaminants. Nonetheless, instruments created by organizations such as WHO and EFSA are increasingly being used to evaluate their exposure and ensure food safety [7,8,73]. Nevertheless, establishing the levels of contaminants for each individual product or even for individual regions remains a considerable challenge. Therefore, CONT11 was used. CONT11 is one of the few food composition databases currently available that evaluates food contaminants generated during thermal processing, making this study particularly relevant. Although the data obtained were substantial, the use of the FFQ may have overestimated dietary intake. Moreover, it is important to note that the application of FFQs for the analysis of some of these contaminants is subject to an additional limitation, specifically the inability to record the method of thermal processing adequately. Consequently, contaminant values must be estimated at intermediate values. Conversely, the implementation of 24 h intake records, which include detailed cooking method specifications for each food item, would facilitate the association of these records with exposure data, resulting in a more precise estimation of exposure levels. Nonetheless, the FFQ utilized, despite not having undergone specific validation for pregnant women, has demonstrated adequate validity and reproducibility in other populations, including adult and child populations in different geographical regions, which enables it to be identified as a robust instrument [32,74]. Another limitation of the present study is the number of pregnant women included in the analysis. In order to strengthen and confirm the results, it would be necessary to include a larger sample size in future research; for example, in our regression analyses, the covariates exerted a considerable influence on the model, and the relatively small sample size may have been a limiting factor. Although the results obtained are consistent with the scientific literature, the significant associations observed remain somewhat uncertain. Future studies should include biomarker analyses to corroborate these findings and more information about the environment and different additional parameters, such as lifestyle, in order to deepen and design more effective health prevention actions. Nonetheless, this study is one of the few to evaluate several contaminants from thermal food processing in pregnant women.

## 5. Conclusions

This study highlights the need to monitor exposure to dietary contaminants in pregnant women to protect fetal development and neonatal health. A decrease in contaminant intake was observed during the third trimester, possibly due to increased dietary concerns. The intake of certain dietary contaminants, such as acrylamide or polycyclic aromatic compounds, has been correlated with maternal-fetal adverse effects, such as reduced birth weight and other anthropometric parameters, highlighting the importance of exposure reduction. Tailored reduction strategies targeting specific food groups identified as major contributors to contaminant exposure may be more effective. Consequently, the results of this research provide a basis for future investigations.

## Figures and Tables

**Table 1:** Characteristics of mother-child pairs (*n* = 84).

Characteristic	%, Mean ± SD
**Maternal Characteristics**	
Maternal age	34.9 ± 5.7
Weight (Kg)	68.2 ± 14.7
BMI (kg/m^2^)	23.7 ± 5.6
Energy intake	2419 ± 386
**Infant Characteristics**	
Infant sex (male/female)	46.4%/53.6%
Gestational age (weeks)	39.2 ± 1.92
Birth weight (g)	3277 ± 508
Height of the newborns (cm)	50.3 ± 2.68
Head circumference (cm)	34.1 ± 1.23

**Table 2:** Intake and exposure to all contaminants of pregnant women in the study.

Contaminants	Mean ± SD Intake (*/day)	Mean ± SD Exposure (*/kg body weight/day)
HMF5 (mg)	64 ± 72.7	0.94 ± 1.06
PYRR (mg)	12.4 ± 7.7	0.18 ± 0.12
AMCOMP (mg)	67.4 ± 48.5	1.1 ± 0.69
FUROS (mg)	23.4 ± 15.8	0.3 ± 0.22
ACRYL (µg)	70.3 ± 42.4	1.08 ± 0.7
FURAN (µg)	14.5 ± 7.8	0.22 ± 0.1
NITRA (mg)	139 ± 67	2.08 ± 0.97
NITRI (mg)	11.3 ± 5.5	0.15 ± 0.07
NITRN (µg)	6.57 ± 3.04	0.10 ± 0.04
B(A)P (µg)	0.64 ± 0.17	0.04 ± 0.006
TPAHC (µg)	4.7 ± 1.1	0.07 ± 0.04

The “*” is equivalent to the units of mg or µg, depending on the contaminant.

## Data Availability

The data presented in this study are available on request from the corresponding author. The data are not publicly available due to confidentiality concerns.
